# Assessment of Air Pollution Aggravation during Straw Burning in Hubei, Central China

**DOI:** 10.3390/ijerph16081446

**Published:** 2019-04-24

**Authors:** Bo Zhu, Yu Zhang, Nan Chen, Jihong Quan

**Affiliations:** 1 Hubei Environmental Monitoring Centre, Wuhan 430079, China; zhubo125@whu.ehu.cn (B.Z.); chennan2000@hotmail.com (N.C.); quanjih@163.com (J.Q.); 2 School of Remote Sensing and Information Engineering, Wuhan University, Wuhan 430079, China

**Keywords:** air pollution, Central China, particulate matter, straw burning

## Abstract

Crop straw burning frequently occurs in Central China, where agriculture is highly productive. We carried out a two-month observation on straw burning in Hubei Province from September 1 to October 31, 2015 to track the variations of air pollutants and comprehensively quantify their influence on regional air quality. Results showed that the concentration of suspended particles (particles smaller than 2.5 or 10 µm, i.e., PM_2.5_/PM_10_) and gas pollutants including ozone (O_3_), sulfur dioxide (SO_2_), nitrogen dioxide (NO_2_), and carbon monoxide (CO) was significantly enhanced with the increasing number of fire spots. The average daily concentrations of PM_10_, PM_2.5_ and O_3_ during the intensive burning period (from October 12 to 25) exceeded the daily limits published by the World Health Organization (WHO) by 101.8, 72.7 and 59.1 μg/m^3^, respectively. In the hourly distribution of pollutant concentration, PM_10,_ PM_2.5_, O_3_, SO_2_, NO_2_ and CO were 63.49%, 46.29%, 65.56%, 64.40%, 48.57% and 13.49% higher during burning periods than during non-burning periods. Statistical results based on the air quality index (AQI) indicated that biomass burning was the key factor for the deterioration of local air quality, with a contribution ratio exceeding 41%. Additionally, the pollutants were more spatially homogeneous during the burning period than during the non-burning period. Straw burning not only worsened the local air quality but also raised the pollution level of surrounding regions due to the transport of air mass.

## 1. Introduction

Exposure to air pollution is a key risk factor for human health, causing a large burden of disease [1]. Biomass burning is an important contributing source to air pollution [2,3,4,5,6,7,8,9,10]. Naeher, et al. (2007) [11] reported that agricultural fires emit significant quantities of known health-damaging pollutants, including several carcinogenic compounds. Many studies have documented the health impacts of exposure to these gases [12]. Therefore, biomass burning is arousing considerable public concern. Existing studies revealed that fine particulate matter (particles smaller than 2.5 µm, i.e., PM_2.5_) could be greatly affected by the smoke generated by biomass burning [2,3,13,14,15]. Cheng, et al. (2014) [3] examined the effect of agricultural waste burning on urban air quality in Shanghai, China and found that overall PM_2.5_ concentration increased from 82 μg/m^3^ to 144 μg/m^3^.

In addition to particles, the smoke emitted by open burning can also affect trace gas concentration [14,16,17] remarkably. In Russia, ozone (O_3_) concentration increased from 59.7 µg/m^3^ to 86.6 µg/m^3^, nitrogen dioxide (NO_2_) from 2.52 µg/m^3^ to 6.12 µg/m^3^ and sulfur dioxide (SO_2_) from 1.86 µg/m^3^ to 5.69 µg/m^3^ during the burning period [14]. Meanwhile, minimal increases in O_3_, SO_2_, NO_2_, and carbon monoxide (CO) and nitric oxide (NO) concentrations were detected by Kim, et al. (2005) [18] in Los Angeles, Southern California wildfires in 2003. In Brisbane, Australia, He, et al. (2016) [16] studied the spatial variation of gas pollutants, and they found that NO_2_, CO and SO_2_ showed positive correlation and spatial homogeneity during the burning period, which was not observed during the non-burning period. Additionally, Cheng, et al. (2014) [3] found that the boundary layer height (BLH) was only 240–399 m during the burning period, and the stagnant weather condition enhanced the accumulation of air pollutants. Xue, et al. (2014) [19] and Wu, et al. (2017) [20] investigated the transport of the emitted smoke by the Hybrid Single Particle Lagrangian Integrated Trajectory (HYSPLIT) model, and confirmed its influence on the air quality in the transport pathways based on in-situ observations.

Straw burning annually occurs in Central China, which is an important grain-producing region in the country. However, a comprehensive impact analysis of smoke emitted by biomass combustion on air quality in the area has been scarcely quantified. This type of analysis is important to regional pollution control. In this study, we focused on pollutant emissions from straw burning during the harvest season (September and October) in Hubei Province, Central China and demonstrated their effects on air quality. This work analyses the daily variations of particulate matter, gaseous pollutants and meteorological factors during the entire episode, quantifies the effect of open biomass burning on local air quality, evaluates the spatial homogeneity of pollutants among monitoring sites and identifies the impact of biomass burning on the surrounding areas.

## 2. Materials and Methods 

**Figure 1:**
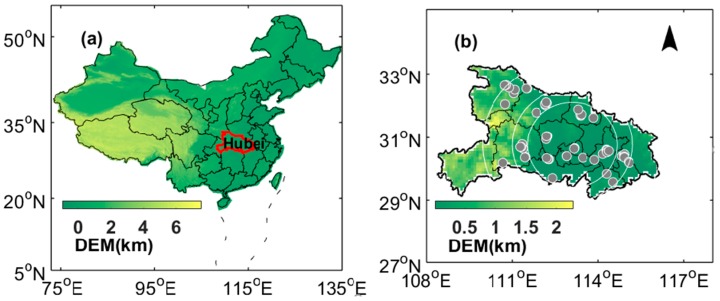
(**a**) Location of Hubei province (the region circled by the red line) in China. (**b**) Location of the monitoring sites (grey dots). The white arc represents the buffer (150, 150–200 and 200–300 km).

Hubei Province lies in Central China and belongs to the midstream of Yangtze River. This province covers an area of 185,900 km^2^ and supports more than 58 million people [21,22]. The location of Hubei Province is shown in Figure 1a. The gradients of color (green/yellow) represents a variation in elevation based on the digital elevation model (DEM) provided by the United States Geological Survey (USGS) with a resolution of 90 m. This province is one of the largest commercial grain production bases in China. Open biomass burning occurs frequently in the autumn harvest season from September to November. In this study, we collected air quality monitoring data containing concentration measurements of solid and gas pollutants, air quality index (AQI), and satellite-based aerosol optical depth (AOD) to characterize and assess the effect of biomass burning on air quality.

### 2.1. Materials

#### 2.1.1. Straw Burning Information

Satellite remote sensing technology, an effective way of monitoring open biomass combustion, has been widely used for obtaining active fire information [9,19]. The brightness temperature of the thermal infrared band obtained from the Moderate Resolution Imaging Spectroradiometer (MODIS) can be used to monitor regional thermal anomalies, which made MODIS an effective method for the monitoring of fire spots [23,24]. In this work, the daily fire spot dataset was derived from the MODIS Fire Information for Resource Management System developed by National Aeronautics and Space Administration (NASA) (https:// irms.modaps.eosdis.nasa.gov/), with a spatial resolution of 1 km.

However, the locations of fire spots obtained from satellite may contain deviations. Field investigation shows that straw burning usually occurs in the afternoon or evening, while the transit time of MODIS is 10:30 a.m. (Terra) or 1:30 p.m. (Aqua) in local time. Thus the monitoring accuracy for fires, especially those with low combustion intensity would be limited. In addition, passive sensors are susceptible to cloud. In order to verify the accuracy of satellite-based fire spot location, we used the unmanned aerial vehicle (UAV) to re-check these fire spots, with a total of 18 flights over the experimental area from September 1 to October 31, 2015. The distance deviations of fire spots monitored by UAV and MODIS are shown in Table 1. The accuracy of fire spots obtained from satellite remote sensing can reach 82.6% with a distance error less than 3 km. Removing the fire spots with a distance deviation of more than 3 km, and the number of fire spots in September, October and November is 60, 206 and 1, respectively. The number of fires is 158 between October 12th and October 25th, which is defined as the intensive burning period here.

#### 2.1.2. Sampling Site and Ambient Monitoring

PM_2.5_, PM_10_, O_3_, SO_2_, NO_2_, CO and meteorological factors, including temperature, relative humidity (RH), pressure and wind speed, during the episode were synchronously monitored at sampling sites in Hubei Province, as shown in Figure 1b. All datasets covered from September 1 to October 31, 2015. The details of experimental instruments for major pollutants are shown in Table 2.

#### 2.1.3. Himawari-8 AOD Data

AOD product at 550 nm were obtained from Himawari-8, which was launched by the Japan Meteorology Agency (JMA) in October 2014. It carries the Advance Himawari Imager (AHI), which has 16 bands from visible to infrared wavelengths and different spatial resolutions. The spatial resolution and temporal resolution of AOD data used in this study are 0.05° and 1 h [5,25]. 

### 2.2. Methods

#### 2.2.1. Correlation and Homogeneity Analysis

The effects of straw burning on local air pollution were evaluated on the basis of two statistical parameters, namely, the correlation coefficient (*R*) and coefficients of divergence (COD). Parameter R can be used to assess the similarity of pollutant sources among different sites, while COD indicates the homogeneity of spatial distribution of pollutants. The *R* is calculated by Pearson’s formula as follows:

$$R_{fh}=\frac{\sum_{i=1}^{n}\left(y_{if}-\bar{y_{f}})(y_{ih}-\bar{y_{h}}\right)}{\sqrt{\sum_{i=1}^{n}{\left(y_{if}-\bar{y_{f}}\right)}^{2}\sum_{i=1}^{n}{\left(y_{ih}-\bar{y_{h}}\right)}^{2}}},$$

where *n* is the number of sampling sites, *f* and *h* are two different sampling sites, $y_{if}$ is the value in the *i*th measurement at site *f*, $\bar{y_{f}}$ is the average of the sample reference values at site *f*, $y_{ih}$ is the corresponding value of the *i*th measurement at site *h* and $\bar{y_{h}}$ is the average of the sample reference values at site *h*. According to He, et al. (2016) [16], more positive correlation among the stations could be indicative of a widespread air pollution event covering the entirety of the air quality monitoring network. In this case, it could be considered that the pollutants observed by local stations had a similar or same emission source to the large extent.

The COD was previously used in the research of reference [18], and the calculation formula is as follows:

$${COD}_{fh}=\sqrt{\frac{1}{n}{\sum_{i=1}^{n}\left(\frac{X_{if}-X_{ih}}{X_{if}+X_{ih}}\right)}^{2}},$$

where *n* is the number of observations, *f* and *h* are two different sampling sites, and $X_{if}$ is the *i*th measurement at site *f*; the corresponding $X_{ih}$ is the corresponding observation at site *h*. Previous studies indicated that the value of COD less than or equal to 0.20 indicates the relatively homogeneous distribution of pollutants [16].

*R* and COD of each pollutant (PM_10_, PM_2.5_, NO_2_, O_3_, CO and SO_2_) were calculated among different sites in burning and non-burning periods, respectively. Furthermore, the correlation between pollutants and fire spots was further investigated during the intensive burning period on the basis of the buffer analysis. The buffer, positioned in the geometric center of all fire spots, was set to three ranges, namely, 150, 150–200 and 200–300 km, as shown in Figure 1b.

#### 2.2.2. Evaluation of Secondary Aerosols’ Generation

According to Lin. (2002) [26], the nitrogen oxidation ratio (NOR) and sulfur oxidation ratio (SOR) can evaluate the conversion degree of NO_2_ to NO_3_^−^ and SO_2_ to SO_4_^2−^, which is the major source of secondary inorganic aerosols. SOR and NOR can be calculated as follows [26,27,28,29]:

$$SOR=\frac{n\left(SO_{4}^{2-}\right)}{n\left(SO_{4}^{2-}\right)+n\left(SO_{2}\right)},$$

$$NOR=\frac{n\left(NO_{3}^{-}\right)}{n\left(NO_{3}^{-}\right)+n\left(NO_{2}\right)}.$$

where *n*(*NO*_3_^−^), *n*(*NO*_2_), *n*(*SO*_4_^2−^), *n*(*SO*_2_) is the molar concentration of particulate nitrate, gas-phase NO_2_, particulate sulfate and gas-phase SO_2_, respectively.

#### 2.2.3. Evaluation of Meteorological Contribution

Assuming that other emissions remain the same except for straw burning over time, and a meteorology-driven anomaly factor (indicated by A here) is defined to evaluate the contributions of the meteorological variety to pollutants. The formula definition is as follows, referring to reference [30].

$$A=\frac{\frac{1}{n}\sum_{j=1}^{n}\left(PM_{j}-\bar{PM_{non\text{-}burning}}\right)}{\frac{1}{m}\sum_{i=1}^{m}\left(PM_{i}-\bar{PM_{non\text{-}burning}}\right)},$$

where *n* and *m* are the number of days under the condition of PBL < 500 m and wind speed < 2 m/s during the non-burning and intensive burning period, respectively; *PM* represents the concentration of PM_2.5_, $\bar{PM_{non\text{-}burning}}$ is the mean value of PM_2.5_ during the non-burning period. The denominator can represent the composite anomaly due to the effects of both meteorology and emissions to some extent, while the numerator represents the “meteorology-driven” anomaly.

#### 2.2.4. Pollutant Transport Analysis

The HYSPLIT model, developed by National Oceanic and Atmospheric Administration Air Resources Laboratory (NOAA ARL), is widely used for the tracking and analysis of air mass trajectories based on the regional meteorological data [31]. In order to evaluate the effect of straw burning in Hubei Province on the air quality in surrounding areas, the forward transport pathways of airflows during the intensive burning period were calculated, centered the geometric centroid (113.09° E, 30.76° N) of the fire spots at height of 1000 m above ground level (to avoid the influence of terrain undulation) [32]. The meteorological data adopted in this study was from the NCEP Global Data Assimilation System (GDAS), with a spatial resolution of 0.5° × 0.5°.

## 3. Results

### 3.1. Daily Variation of Pollutants and Meteorological Conditions

**Figure 2:**
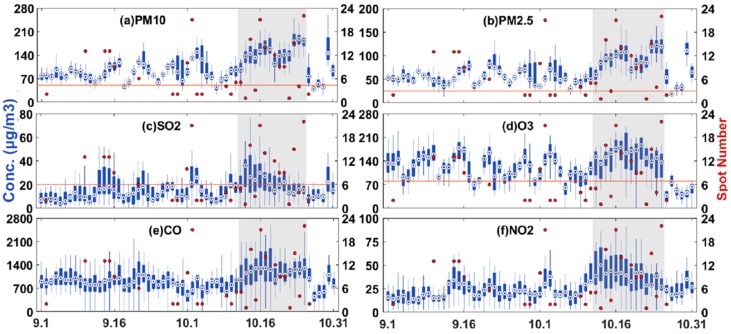
24-h average concentrations of PM_10_ (**a**), PM_2.5_ (**b**), SO_2_ (**c**) O_3_(**d**), CO (**e**) and NO_2_ (**f**) and daily maximum 8-h average concentration of O_3_ (**c**) at all monitoring stations from September to October 2015. The orange horizontal lines represent the WHO guideline for 24-h PM_10_, PM_2.5_ and SO_2_ and 8-h O_3_, which are 50 µg/m^3^, 25 µg/m^3^, 20 µg/m^3^, 100 µg/m^3^, respectively. The red point in each subplot represents the number of fire spots for each day.

The 24-h average concentrations of PM_10_, PM_2.5_, SO_2_, NO_2_ and CO and daily maximum 8-h average concentration of O_3_ were obtained from monitoring stations from September to October 2015. The daily variations of the concentrations of the six pollutants are presented in Figure 2. The red point in each subplot represents the number of fire spots. In accordance with the presence or absence of fire spots, we divided the whole experiment into a burning and non-burning period. The intensive burning period is from 12 to 25 October 2015, which is emphasized by the grey color in the figure. In summary, the daily concentrations of all the pollutants were higher during the burning period. The concentration of each pollutant increased with the increasing number of fire spots. The daily mean concentrations of PM_2.5_ and PM_10_ at almost all sites were higher than the concentrations recommended by the World Health Organization (WHO) guideline (24 h). As shown in Figure 2c–f, the concentration variations of O_3_, SO_2_, NO_2_ and CO were consistent with biomass burning occurrence during the entire study period.

A remarkable increasing trend can be observed for all pollutants during the intensive burning period. Specifically, the daily average concentration of PM_10_ and PM_2.5_ peaked (151.8 μg/m^3^ and 97.77 μg/m^3^ respectively), which exceeded the WHO limits by 101.8 µg/m^3^ and 72.7 µg/m^3^, respectively. The daily maximum 8-h average concentration of O_3_ in September was rather high because of the high ambient temperature, which would facilitates the generation of ozone by chemical reactions [13,16,33,34]. The average daily concentration of O_3_ exceeded the WHO guideline by 59.1 µg/m^3^ during the intensive burning period, and that of SO_2_ was up to twofold higher than the WHO guideline. The concentration of CO and NO_2_ also increased during the intensive burning period.

**Figure 3:**
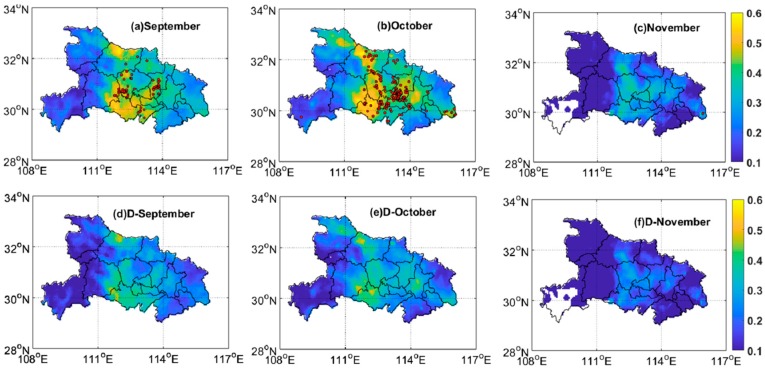
Average distributions of AOD (from (**a**) to (**c**)) and the corresponding difference (from (**d**) to (**f**)) with the mean value of AOD over two years (July 2015 to July 2017) in Hubei Province. The red point in each subplot represents the location of fire spot for each month.

The spatial distributions of monthly average AOD from September to November are shown in Figure 3. The overall spatial distribution of AOD was reasonably consistent with the distribution of the fire spots. The monthly Himawari-8 AOD reached the maximum level as the number of fire spots peaked in October, as shown in Figure 3b, with the average value being greater than 0.5. In order to further confirm the effect of straw burning on aerosols in local areas, the corresponding difference with the mean value of AOD over two years (July 2015 to July 2017) was calculated, as shown in Figure 3d–f. The results showed that straw burning obviously enhanced regional AOD.

**Figure 4:**
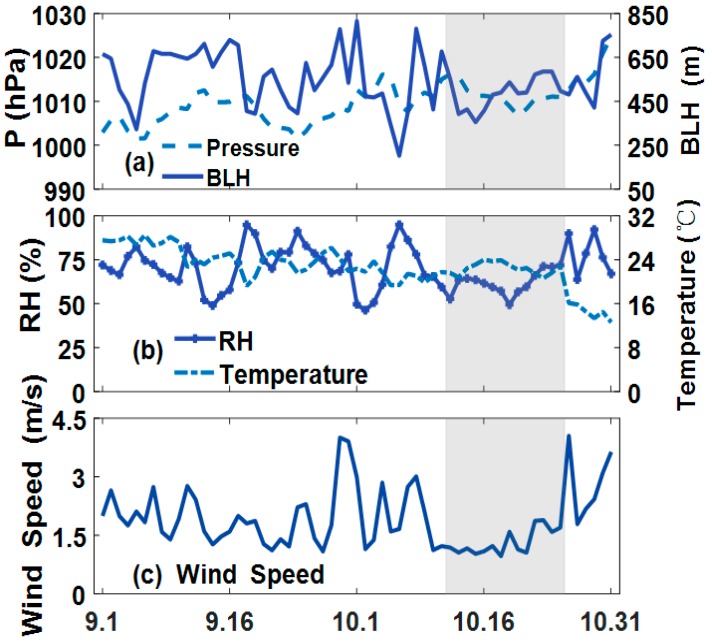
Variation of meteorological factors (Pressure, BLH, temperature, RH and wind speed) in the study area from September to October 2015.

Temporal variations in meteorological parameters, including BLH, pressure, RH, temperature and wind speed, are shown in Figure 4. In the entire period, BLH was in the range of 235–760 m, and pressure varied between 1003 and 1026 hPa. Temperature range was 13–29 °C with RH ranging from 50% to 95%. A summary of the statistical results for each meteorological parameter in the non-burning, burning and intensive burning periods is shown in Table 3. Evidently, the average meteorological conditions during burning and non-combustion were relatively consistent, except RH. This finding verified that the increase in local pollutants during burning period was largely caused by straw burning rather than meteorological conditions. Notably, the increase in all pollutants in the air led to the deterioration of meteorological conditions during the intensive burning period. The statistical data indicated that BLH reached its minimum value and stabilized at approximately 490 m. Moreover, the average wind speed sharply declined to 1.3 m/s during the intensive burning period. Pollutants peaked in the period under adverse dispersion and stagnant weather conditions. The fraction of “meteorology-driven” anomaly in the enhanced PM_2.5_ is 0.121, which suggest that meteorological variety had an impact on the aggravation of pollution, but the main reason is still the high emissions from straw burning. 

### 3.2. Diurnal Patterns of Pollutants for Burning and Non-Burning Periods

**Figure 5:**
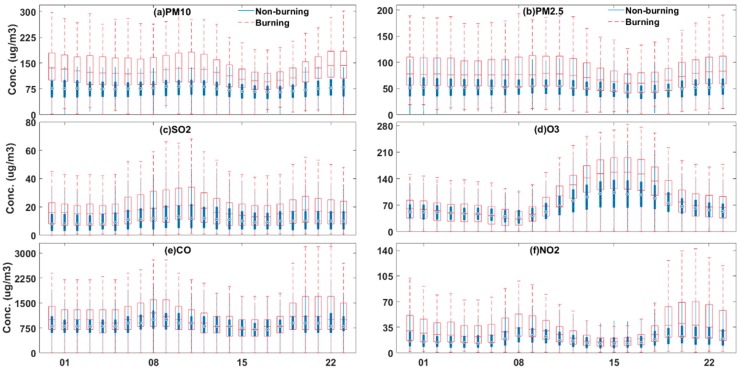
Examples of the average diurnal variation of PM_10_ (**a**), PM_2.5_ (**b**), SO_2_ (**c**) O_3_ (**d**), CO (**e**) and NO_2_ (**f**) concentrations during the Burning and Non-burning periods.

Diurnal variations in PM_10_, PM_2.5_, O_3_, SO_2_, NO_2_ and CO concentrations during the burning and non-burning periods are presented in Figure 5. In the diurnal pattern, the concentrations of pollutants were higher during the burning period than those in the non-burning period. Specifically, the hourly average concentrations of PM_10_, SO_2_ and PM_2.5_ were 47.83, 23.34 and 5.76 μg/m^3^ higher than those in the non-burning period. However, a slight increase (13%) in CO concentration relative to the average concentration during the non-burning period was found, which is different from that reported by previous research [16,35]. A combustion process takes two forms, i.e., smoldering and flaming, and generally begins with smoldering, and then become flaming combustion in good ventilation. According to reference [36], in flaming combustion, the ratio CO to CO_2_ is remarkably small because more volatile CO was combusted as a result of higher oxygen availability. Conversely, in smoldering combustion, CO could not be combusted effectively because of low oxygen availability and low temperature. Therefore, the process of combustion usually contended with the CO_2_ or CO competition, flaming or smoldering, and it would not show a linear relationship on the emission of pollutants. In this study we just found a small increase in carbon monoxide, which should be because more CO was oxidized. Notably, the O_3_ concentration remarkably increased during daytime and decreased at night during the burning and non-burning periods. By contrast, the amount of O_3_ varied over the two periods. Specifically, the increase of O_3_ concentration in the daytime (i.e., 10:00 a.m. to 19:00 p.m.) was evidently higher during the burning than the non-burning period. Overall, the NO_2_ concentration showed an opposite trend to O_3_. This opposite diurnal patterns for O_3_ and NO_2_ found in this study are similar to those reported by He, et al. (2016) [16] and Wonaschutz, et al. (2011) [34]. This phenomenon is attributed to the large amount of NO_2_ produced by biomass burning, which would promote photolysis reactions by consuming NO_2_ and hence generate more ozone in the afternoon [16]. However, the chemical reaction phases out owing to the absence of sunlight in the night time, and ozone cannot be produced from the precursors [33]. By contrast, O_3_ participates in the titration reaction (the reaction of O_3_ with NO to NO_2_ and O_2_) [13,34] to produce NO_2_. Therefore, the extent of increase in NO_2_ concentration during the biomass burning period is higher at weak light.

The average concentrations of PM_10_, PM_2.5_, SO_2_, NO_2_, O_3_, CO and AQI during the burning and non-burning periods are presented in Table 4. The PM_10_, PM_2.5_, SO_2_, NO_2_, O_3_ and CO concentrations increased by 63.49%, 46.29%, 65.56%, 64.40%, 48.57% and 13.49%, respectively, after open biomass burning. The values are based on the hourly average concentrations. We used the pollution index of AQI to assess the extent of air quality deterioration. The statistical data indicated that the open biomass burning deteriorated the local air quality to a large extent (41.9%).

### 3.3. Spatial Variations of Air Pollutants

#### 3.3.1. Overall Spatial Variations

**Figure 6:**
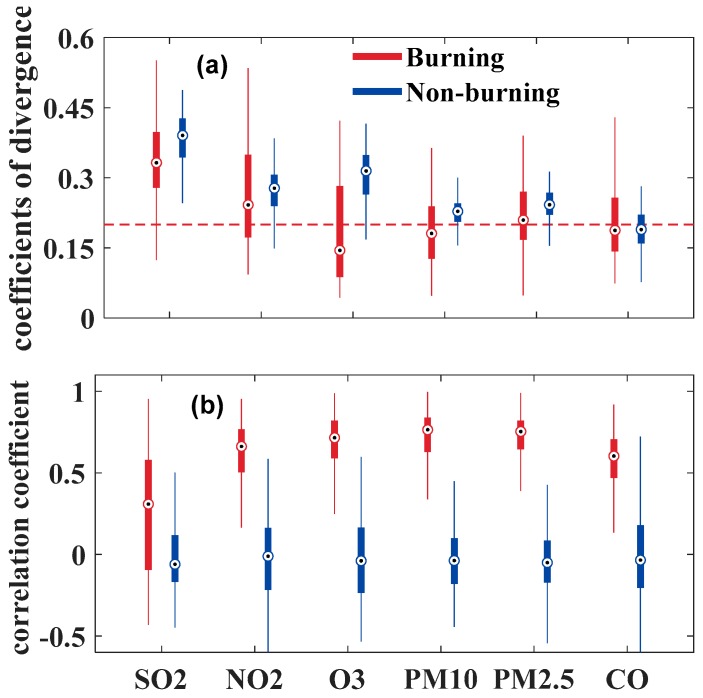
Overall inter-site COD (**a**) and *R* (**b**) of SO_2_, NO_2_, O_3_, PM_10_, PM_2.5_ and CO for the burning and non-burning periods. The horizontal dotted line indicates the COD value of 0.20 in subplot (**a**).

The *R* and COD values in all the monitoring stations were calculated. The results were summarized as box–whisker plots in Figure 6. The calculated CODs exhibited lower values during the burning period than in the non-burning period, except for CO. Specifically, COD analysis revealed mostly homogeneous spatial distribution of O_3_ concentrations in all inter-sites, which reached the value of 0.15 according to the median of the box–whisker plots during the burning period. Additionally, CODs of PM_10_, PM_2.5_ and CO tended to approach 0.2 during the burning period, thereby indicating relatively uniform spatial dispersions [35]. The *R* values considerably increased during the burning period. The high *R* values during the burning period indicated that all monitoring sites were largely influenced by the similar pollution source.

**Figure 7:**
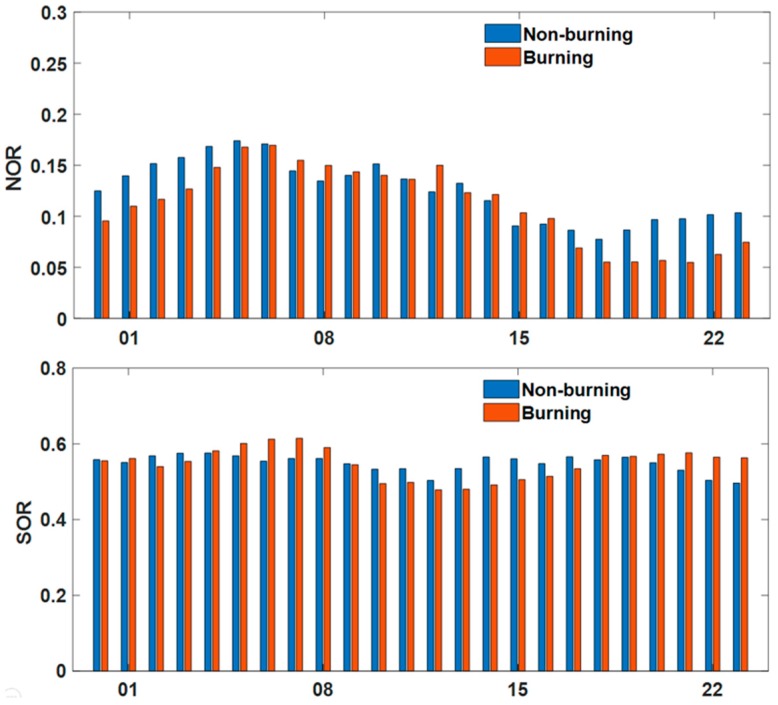
Diurnal variation of SOR and NOR during Burning and Non-burning period.

To further determine whether the spatial homogeneity enhancement of pollutants was caused by emissions from straw burning or generated secondary aerosols, the diurnal variation of SOR and NOR during burning and non-burning periods were investigated, as shown in Figure 7. The average SOR (NOR) were 0.548 (0.113) and 0.549 (0.124) during the burning and non-burning, respectively. Previous studies [37,38] have reported that the value of SOR is generally less than 0.10 in the primary pollutant, and higher SOR and NOR means more secondary aerosols of sulfates and nitrates are generated through photochemical oxidation in the atmosphere [26,39]. Therefore, it can be concluded that there were significant secondary aerosol formation in both burning and non-burning stages, and the chemical conversion rates in the two stages were similar. This result indicates that the formation of secondary aerosols has little influence on the variation of COD and *R* values, which further proves that combustion emissions promoted the homogeneity of pollutants among different monitoring stations.

#### 3.3.2. Spatial Variations along with the Distance Buffer

**Figure 8:**
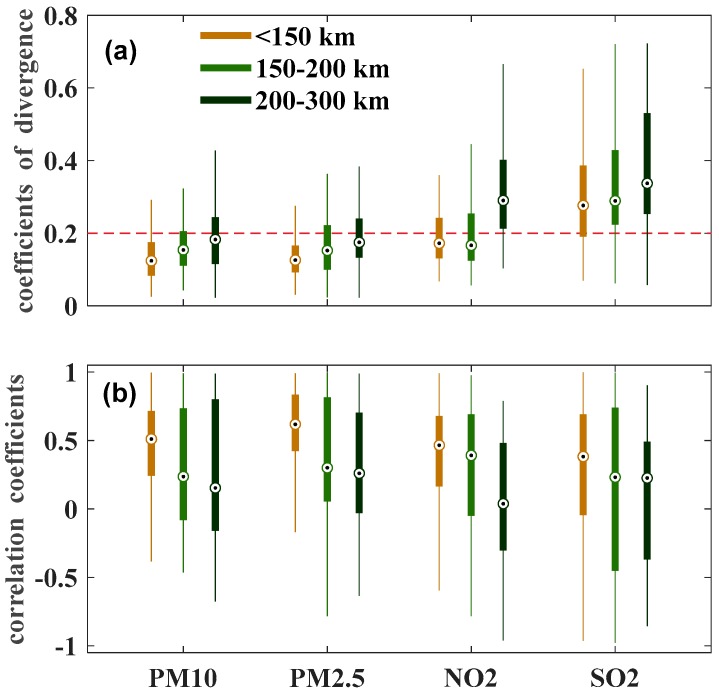
Overall inter-site CODs (**a**) and *R* (**b**) of SO_2_, NO_2_, O_3_, PM_10_, PM_2.5_ and CO in different buffers during the intensive burning period.

We selected three distance buffers to further analyze the spatial variations during the intensive burning period and calculated CODs and R for PM_10_, PM_2.5_, NO_2_ and SO_2_. The results are shown in Figure 8. The CODs increased, and *R* correspondingly decreased with the increase of distance. In the distance buffer of 150 km, the CODs of all pollutants were lower than 0.2, except SO_2_, thereby indicating a strongly homogeneous spatial distribution, especially for PM_2.5_ and PM_10_. As for *R*, the associations with the inter-sites were much stronger in the distance buffer of 150 km than 150–200 and 200–300 km.

These results above mentioned show that open biomass burning increased the spatial homogeneity for pollutants. Pollutant concentrations in the stations showed more positive correlations during the burning period than in the non-burning period.

### 3.4. Transport of Pollutants and Regional Effects

**Figure 9:**
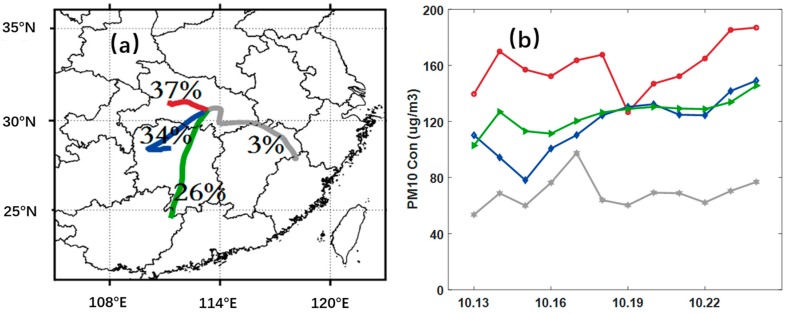
(**a**) Examples of the air mass analysis for October 13–25, 2015. The percentage for each trajectory represented the air mass contributions. (**b**) PM_10_ concentrations on the monitoring sites corresponding to each trajectory line. The four colored trajectories in (**a**) correspond to each line in (**b**).

Cluster analysis of the 36-h forward trajectories was conducted using the HYSPLIT model to further identify the effect of open biomass burning on air quality in the surrounding areas. The 13-day (from 12 to 25 October 2015) forward trajectories were calculated, centered the geometric centroid (113.09° E, 30.76° N) of all fire spots during the period at the height of 1000 m above ground level. The forward trajectories were grouped into four different clusters. The monitoring stations were relatively uniformly selected according to the length of each cluster. Forward trajectories and daily concentrations of PM_10_ corresponding to the trajectory line are shown in Figure 9.

During the intensive burning period, particulate mass concentrations at all monitoring sites in the transport pathways showed varying degrees of increase. The main airflow affected by the north wind mainly blows down to the south, thereby mixing with the pollutants produced by open biomass burning along the path. Approximately 37% of air masses flowed northward from the fire spot center and reached the central and western parts of Hubei Province after a short distance transport. The maximum concentration of PM_10_ at the monitoring sites around the red trajectory reached 200 µg/m^3^. The daily average PM_10_ concentrations around the blue path (to the southwest) increased from 70 µg/m^3^ to 160 µg/m^3^ owing to the static atmospheric dynamic conditions, followed by the green trajectory (to the south) in Hunan Province, which had a peak concentration of 145 µg/m^3^. Meanwhile, the increases in southeast monitoring stations around the grey trajectory were relatively small because of the small air mass (3%).

## 4. Conclusions

Six types of air pollutants were analyzed in this study to confirm the effect of straw burning on air quality from September 1 to October 31, 2015 in Hubei Province. The results obtained here were comparable with the previous studies, and the rising trend of pollutants over monitoring stations during the experimental period was comparable to observations in references [16,17]. The key findings in this paper are summarized as follows:

(1) During the intensive burning period, the air quality significantly deteriorated, and PM_10_, PM_2.5_ and O_3_ exceeded the WHO limits by 101.8, 72.7 and 59.1 µg/m^3^, respectively. Poor meteorological conditions, indicated by a stable high pressure, low wind speed and low BLH, further enhanced the pollution level during the intensive burning period.

(2) In the diurnal patterns of pollutants, the results presented that the hourly concentrations of PM_10_, PM_2.5_, O_3_, SO_2_, NO_2_ and CO caused by open biomass burning increased by 63.49%, 46.29%, 65.56%, 64.40%, 48.57% and 13.49%, respectively. The average AQI during the burning and non-burning periods indicated that open biomass burning deteriorated the local air quality by 41.9%.

(3) The pollutants showed a more positive correlation among sites during the burning period, thereby indicating a highly homogeneous distribution of pollutants. Moreover, straw burning not only worsened the local air quality but also raised the pollution level of surrounding regions due to the transport of air mass.

## Figures and Tables

**Table 1:** Position deviations of fire spots monitored by UAV and MODIS.

Deviations between UAV and MODIS	≤1 km	1–2 km	2–3 km	>3 km
Proportion	39.1%	32.6%	10.9%	17.4%

**Table 2:** List of experimental instruments for major pollutants.

Pollutant	Instrument	Method	Sampling Interval
PM_2.5_	TH-2000PM	β Ray absorption	5 min
PM_10_	TH-2000PM	β Ray absorption	5 min
SO_2_	MODEL 43i	Pulsed fluorescence	5 min
O_3_	MODEL 49i	Ultraviolet photometry	5 min
NO_2_	MODEL 42i	Chemiluminescence	5 min
CO	MODEL 48i	Gas photometry	5 min

**Table 3:** Statistical summary of the average value for meteorological factors (RH, PBL, pressure, temperature and wind speed) in different periods.

Meteorological Parameters	Non-Burning Period	Burning Period	Intensive Burning Period
RH (%)	78.3	61.8	62.5
PBL (m)	535.4	557.7	491.6
Pressure (hPa)	1008.7	1010.5	1010.5
Temperature (°C)	22.2	22.9	22.5
Wind Speed (m/s)	2.1	1.8	1.3

**Table 4:** Hourly average concentrations and increase of pollutants (PM_10_, PM_2.5_, SO_2_, NO_2_, O_3_ and CO) during the burning and non-burning periods (unit: μg/m^3^).

	Burning Period	Non-Burning Period	Increase
PM_10_	122.72	74.89	63.49%
PM_2.5_	73.52	50.18	46.29%
SO_2_	16.56	10.08	65.56%
NO_2_	26.29	17.10	64.40%
O_3_	84.66	64.60	48.57%
CO	950	830	13.49%
AQI	114.1	80.4	41.9%
